# Brief Parent-Child Substance Use Education Intervention for Black Families in Urban Cities in New Jersey: Protocol for a Formative Study Design

**DOI:** 10.2196/55470

**Published:** 2024-05-09

**Authors:** Ijeoma Opara, Kimberly Pierre, Sandy Cayo, Kammarauche Aneni, Catherine Mwai, Aaron Hogue, Sara Becker

**Affiliations:** 1 Yale University School of Public Health New Haven, CT United States; 2 Irvington Department of Health Irvington, NJ United States; 3 Yale University School of Nursing New Haven, CT United States; 4 Yale School of Medicine New Haven, CT United States; 5 Partnership to End Addiction New York, NY United States; 6 Northwestern School of Medicine Chicago, IL United States

**Keywords:** black families, parents, drug use, prevention, urban community

## Abstract

**Background:**

Substance use continues to remain a public health issue for youths in the United States. Black youths living in urban communities are at a heightened risk of poor outcomes associated with substance use and misuse due to exposure to stressors in their neighborhoods, racial discrimination, and lack of prevention education programs specifically targeting Black youths. Many Black youths, especially those who live in urban communities, do not have access to culturally tailored interventions, leaving a critical gap in prevention. Since family is a well-known protective factor against substance misuse for Black youths, it is essential to create sustainable and accessible programming that incorporates Black youths’ and their families’ voices to develop a suitable prevention program for them.

**Objective:**

We aim to understand the cultural and environmental level factors that influence substance use among Black youths and develop a prevention program to increase parent-child substance use education among Black families.

**Methods:**

This study will take place within urban cities in New Jersey such as Paterson and East Orange, New Jersey, which will be the main study sites. Both cities have a large population of Black youths and this study’s team has strong ties with youths-serving organizations there. A formative, qualitative study will be conducted first. Using the first 3 steps of the ADAPT-ITT (Assessment, Decision, Adaptation, Production, Topical Experts, Integration, Training, and Testing) framework we begin the development of an intervention for Black families. Three aims will be described: aim 1, collect qualitative data from Black parents and youths aged 11-17 years from parent-child dyads (N=20) on the challenges, barriers, and facilitators to communicating about substance use; aim 2, adapt a selected evidence-based intervention for Black families and develop a family advisory board to guide the adaptation; and aim 3 assess the feasibility of the intervention through theater testing, involving the family and community advisory board.

**Results:**

This study is part of a 2-year research pilot study award from the National Institutes of Drug Abuse. Data collection began in May 2023, and for aim 1, it is 95% complete. All aim 1 data collection is expected to be complete by December 30, 2023. Data analysis will immediately follow. Aim 2 activity will occur in spring 2024. Aim 3 activity may begin in fall 2024 and conclude in 2025.

**Conclusions:**

This study will be one of the few interventions that address substance use among youths and uses parents and families in urban communities as a protective factor within the program. We anticipate that the intervention will benefit Black youths not only in New Jersey but across the nation, working on building culturally appropriate, community-specific prevention education and building on strong families’ relationships, resulting in a reduction of or delayed substance use.

**International Registered Report Identifier (IRRID):**

DERR1-10.2196/55470

## Introduction

### Significance of This Study

While Black youths have lower rates of substance use than White and Hispanic youths, Black youths tend to have worse outcomes associated with substance use such as poor academic outcomes, involvement in the criminal justice system, and engaging in sexual risk behaviors while using drugs [1]. Despite the well-established association between parent-child communication and adolescent substance use, racial-specific differences among Black families compared to other racial-ethnic groups in parent-child drug use communication have received limited attention in intervention development. Thus, the adaptation of a race-specific parent-child substance use education intervention for Black families which can promote family bonding, communication, and supervision, as well as acknowledging racial and ethnic-specific norms, values, and pride, is necessary. In addition to racial stressors such as racial discrimination that Black families uniquely experience, it is essential for a racial-specific, strengths-based prevention intervention be used and adapted for urban Black youths and their families.

### Background

Decades of research have established substance use as a public health concern among youths [2,3]. According to the National Survey on Drug Use and Health, approximately 24% of Black or African American people aged 12 years or older used illicit drugs in the past year, 10% were classified as having alcohol use disorder, and 10% were underage (aged 12-20 years) when drinking alcohol [4,5]. Whereas Black youths are more likely to start drinking at a later age than their peers and consume less alcohol [6,7], they also tend to experience more negative social consequences due to drinking compared to White youths [2,8,9]. Additionally, Black youths experience greater negative consequences from drug use, such as marijuana, compared to young men in other racial and similar socioeconomic status categories. Such consequences include increased accidents, illnesses, injuries, lower recovery rates from alcohol dependency, greater interpersonal issues, carceral issues, and economic challenges [10-13]. Black youths in urban settings tend to experience a unique set of stressors associated with their families, peers, and environment that in turn impact their mental health, school attendance and engagement, substance use, sexual health, and overall ways they cope with those stressors [14]. In addition to commonly cited risk factors for drug use among Black youths, especially those that live in urban communities such as exposure to community trauma, peer pressure, and neighborhood disorganization, research suggests that attending to the developmental concerns of Black youths also requires addressing the needs of the family unit [14].

### Family as a Protective Factor for Black Youths

Family influences on adolescent development as well as substance use are well documented and the results across the board unsurprisingly point out that parents play a major role when it comes to affecting risk and protective behaviors [1]. In general, the more attentive, engaged, warm, protective, communicative, and close the relationship is, the more it will serve as a protective factor [1,15,16]. Parental bonding plays a protective role against youths using drugs while living in urban environments and parent-child communication is a significant protective factor for youths [15,16]. Our team found that parent-child communication about substance use and the parental modeling and communication of drug use within a neighborhood context were significant factors in reducing drug use and promoting abstinence among Black girls [17]. Other research by our team and others has demonstrated that youths report reduced use of drugs when they have positive relationships with their parents and communicate with their parents about sex [16-18]. However, very little research has specifically focused on family processes involved in drug use prevention among Black families. Salas-Wright et al [19] found that from 2002 to 2016, Black families who had lower socioeconomic status were less likely to discuss substance use education with their children. Most interventions addressing substance use have included large samples of White parents and children, which can result in gaps in prevention research focused on understanding significant cultural differences in family involvement and communication for Black families [16,20,21].

### Challenges Within Black Families

While Black families are not homogenous, many Black youths who have negative consequences of drug use are exposed to traumatic conditions such as poverty, parental unemployment, parental drug use, neighborhood violence, and crime within their environments [22,23]. Within a family context, research has indicated that the more internal and external family stress that youths experience, the more elevated their mental health problems, use of drugs, and sexual risk behavior [7]. In addition, racism that Black youths and families experience can be a risk factor in Black families and a mental health stressor that can lead to substance use [23-26]. Little is known about the intersection of Black parent-child drug use communication and its influence on substance use outcomes in children**.** Consequently, it is essential to understand Black families’ parenting practices and environment context to understand these practices’ effects on youths’ perception of substance use. This will avail the opportunity to create culturally sensitive and tailored interventions specifically designed to meet the unique needs of Black youths. This study protocol describes a race-specific parent-child substance use education intervention adapted for Black families called, “The Development of a Brief Parent-Child Substance Use Educational Intervention for Black Families.” This brief intervention has the potential to guide future substance use interventions focused on Black families with a focus on strength-based and parent-child communication approaches. Considerable studies have validated that participation in culturally tailored family-centered preventative interventions for Black adolescents and their caregivers is associated with offsetting mental health risks, drug use, sexually risky behavior, and academic and behavioral challenges in Black adolescents [27-29]. There is a great need for researchers to develop culturally relevant prevention programs that are designed by and for Black families.

There is only one parent-child intervention that targets risky behaviors (ie, the Strong African American Families program [SAAF]) including substance use for Black families. To be more specific, SAAF is focused on improving family cohesion, dynamics, and communication for Black families and was adapted from the Strengthening Families Program [30]. However, it is 3 hours long and administered in 7 sessions, which may pose barriers described in detail below.

The SAAF program is one of the few prevention programs designed for Black or African American families. The SAAF program is a 7-session program designed for youths aged 10-14 years and their caregivers [30,31]. The goal of SAAF is to build on the strengths of African American families and support parents and youths during the transition from early adolescence to the teen years with a specific emphasis on helping young people avoid risky behaviors (eg, substance use). SAAF has shown positive health outcomes [27,32,33] and has been adapted to include condom education [32] and obesity and eating behaviors [34]. This study seeks to adapt the SAAF intervention to be applied in 2 urban cities in New Jersey: Paterson and East Orange, New Jersey. Both cities are considered urban and have a large population of Black people. This study’s team also has long-standing partnerships with both cities and has experience in recruiting Black youths.

### Barriers to Recruiting Black Families

#### Overview

Traditional family interventions often do not meet the needs of urban Black youths with substance use for a variety of reasons. Overall, parent interventions usually have high attrition [35]. A primary concern with the feasibility of existing parenting interventions is the difficulty ensuring that parents receive an adequate “dose” of the intervention. Adolescent substance use interventions involving parents that have been identified as “well-established” have generally ranged in intensity from 12 to 24 in-person sessions [36]. Further, studies with the highest retention rates of parents, [37] have relied upon home-based sessions or visits, an approach that is challenging to implement within residential facilities that are often short-staffed and financially constrained. Such high-contact interventions may also be difficult for parents of adolescents in substance treatment due to structural barriers related to lower socioeconomic status, such as limited transportation, lack of childcare, and competing demands [38]. Low attendance rates can be the result of busy work schedules and extracurricular activity schedules for youths, as well as a lack of motivation. While SAAF exists, there remain implementation challenges in recruitment for Black parents and children in urban communities. In studies that have used SAAF, a common challenge in scheduling has been mothers’ varied work schedules [30]. Father involvement in the intervention is limited based on several studies that reported on this characteristic [39-41]. Additionally, the youths’ fathers rarely participated in the prevention program even though they were invited to do so [42]. Although SAAF is a model intervention that has shown efficacy in improving knowledge, reducing risky behaviors, and improving mental health outcomes among African American families—there remain implementation challenges. For example, a 7-session intervention may not be feasible for urban Black families who are facing systemic and structural factors and are unable to commit to an intensive, time-consuming schedule. Given the knowledge researchers have on prevention interventions for Black youths and families, community-based approaches must be incorporated as community-based and culturally relevant interventions are the gold standard for urban communities [43]. Hence, the importance of adapting the intervention that can address the documented challenges and reach more Black youths and their parents.

Brief interventions, which are defined as interventions that have 4 or less number sessions, have been emerging as a strong option in substance use, frequently demanding less in terms of time and financial commitment, are one such potential resource for Black families. Brief interventions have shown efficacy in addressing youths’ difficulties such as depression, anxiety, and substance use [44,45]. Specifically of substance use, meta-analytic reviews have found that brief interventions reduce risky drinking [46], and systematic reviews have found that brief-motivation building interventions are well established [36]. Our study aims are as follows:

#### Aim 1

We aim to collect qualitative data from Black parents and youths aged between 11 and 17 years from (N=20) parent-child dyads on the challenges, barriers, and facilitators to communicating about substance use. Recruitment will take place in partnership with community-based organizations and supportive family programs. Semistructured interviews will be conducted with adolescents and their parents to solicit their perspectives on identifiable strategies that have worked to discuss substance use within their family, barriers and facilitators to effective prevention programs, and specific community and cultural norms regarding youths’ substance use.

#### Aim 2

We aim to adapt SAAF intervention using the first 3 phases of the ADAPT-ITT (Assessment, Decision, Adaptation, Production, Topical Experts, Integration, Training, and Testing) implementation framework, phases 1 and 2 which include a family and community advisory board (consisting of 3 parent-child dyads and 3 community leaders) will be developed to guide the adaptation of the intervention.

#### Aim 3

We aim to assess the feasibility of the intervention through theater testing, involving the family and community advisory board, we will enter step 3 of the ADAPT-ITT implementation framework.

## Methods

### Community Profile

#### Paterson, New Jersey

Paterson has a population of roughly 150,000 residents and is the third-largest city in New Jersey [47]. Over 90% of the city’s population identify as either Hispanic (57.7%) or as African American or Black (34.7%) and nearly one-third are foreign-born residents [47]. The principal investigator’s (PI’s) work in the city shows that Paterson youths who drank alcohol during the past 30 days were 3 times more likely to smoke marijuana before being aged 14 years [48]. In terms of accessibility, 60% of Paterson youths purchased alcohol from liquor stores and 40% of Paterson adolescents also admitted to having adults purchase their alcohol from liquor stores [48]. Additionally, the city is currently facing an extreme opioid crisis with Paterson being ranked the 2nd city in New Jersey for the highest rate of heroin overdoses [49].

#### East Orange, New Jersey

East Orange is an urban city also located in Northeastern New Jersey and just 20 minutes away from Paterson. The city has one of the highest rates of drug overdoses in New Jersey and lacks youths’ substance abuse rehabilitation and treatment centers [49]. East Orange also has the highest amount of Black people in the state of New Jersey (85%) [50]. In 2020, a total of 2434 (34%) of Essex County residents admitted for substance abuse treatment were female, 4375 (61%) were Black (non-Hispanic), and 31% of residents were aged younger than 18 years [51]. In our work with the city of East Orange, we conducted a qualitative study in East Orange, New Jersey, with a sample of 45 Black teen girls from the city who participated in focus groups with this study’s team. Themes that arose involved (1) drug use being a major problem in their community, (2) peer pressure and mental health as a major risk factor, and (3) exposure to drugs in specific neighborhoods as an issue.

### Framework for Adaptation

The ADAPT-ITT model is an implementation science framework that guides the adaptation of evidence-based interventions (EBI) for specific settings or populations [39,52]. ADAPT-ITT will be used to adapt the target interventions in partnership with a family and community advisory board, consisting of parents, caregivers, and leaders of family-based organizations in New Jersey.

The framework consists of 8 phases, each of which brings contextual nuances and constraints that determine how the phases are engaged and the timing of corresponding tasks: (1) assess the risk profile of Black families in participating cities: Paterson and East Orange, (2) decide on whether to adopt or adapt an EBI, (3) administer novel methods such as theater testing with families and children to facilitate the adaptation process, (4) plan on what aspects of the EBI need to be adapted and plan on how best to evaluate the adapted EBI, (5) identify additional topic experts to assist in the adaptation process, (6) integrate material from the topic experts to adapt the EBI, (7) train staff to implement the adapted EBI, and (8) test the adapted EBI.

Given the time and financial consideration, we will complete the first 3 tasks at the end of the pilot study. Using the ADAPT-ITT approach, we will first consult with Black families and stakeholders in cities that include a large population of Black people in New Jersey such as Paterson and East Orange to understand their unique challenges in discussing substance use with their children while also seeking their guidance on the development of a racial specific substance use parent-child intervention. A proactive approach, rather than a reactive approach, in the recruitment and retention of minority populations in research is necessary to build trust through community involvement, adapt to cultural norms, develop effective recruitment strategies, and through use of evidence-based practices to engage and retain participants effectively [53]. Therefore, we will use a proactive approach to recruit study participants, which will bring project staff into direct contact with potential participants [53]. This typically involves face-to-face contact with community leaders and organizations, as well as recruitment presentations and meetings in the community.

### Study Design

We will conduct a formative qualitative study design consisting of semistructured individual interviews with (N=20) dyads of Black parents and their children. Qualitative data that will be collected from individual dyad interviews (parent-child) with Black families and children will be used to inform the data. Specifically, the research team will identify themes from data that center around challenges and barriers to drug use education and discussion within the family context and also will ask participants to provide examples of what they would like to see in a family-centered substance use intervention for Black families.

### Eligibility

A parent who identifies as (1) Black, (2) of any age, (3) speaks and understands English, (4) resides in the state of New Jersey, and (5) has a child who identifies as follows. The child must identify as (1) Black, (2) aged between 10 and 17 years, (3) speaks and understands English, and (4) resides in a city that is classified as “urban” in the state of New Jersey. While our study team is primarily focusing on targeting 2 cities: Paterson and East Orange, based on need and our community partnerships, we are also open to including youths and families from other urban cities in New Jersey. The parent and child dyad must meet all criteria to be eligible for this study.

### Ethical Considerations

This study was funded by a larger National Institute on Drug Abuse grant and approved by the Yale University Institutional Review Board in 2022 (2000032674). Due to the sensitivity of the research questions, we requested that written parental consent for youths younger than 18 years be waived for parents and youths and it was granted. All youths who participate in this study will receive a youths’ information sheet, and parents and guardians will receive a parent information sheet. Youths must give verbal consent to participate in this study. We will obtain informed consent from all youths who meet the eligibility criteria and want to participate before they are enrolled in study activities. All participants will be paid US $50.00, in cash, at the end of each interview.

### Aim 1

#### Overview

We aim to collect qualitative data from Black parents and youths aged between 11 and 17 years from parent-child dyads on the challenges, barriers, and facilitators to communicating about substance use.

#### Recruitment

Participation in this study will be strictly voluntary, confidential, and nondiscriminatory. This study will be advertised via posters and flyers located and circulated through community-based organizations, schools, and supportive family programs with which the PI has partnered. In addition, individual interviews with participants will also be recruited using social media sites, including Facebook, Instagram, and Twitter as needed. The PI and her research team are well known in the State of New Jersey and have strong partnerships with cities that have large Black populations such as East Orange and Paterson, New Jersey. During the duration of this study and afterwards, the PI and her research team still intend to conduct workshops and seminars about prevention for youths and girls and dissemination of research about the youths of color and parents specifically. The PI will continue to provide free workshops to youths and their families even if the youths refuse to participate in this study. We will first recruit parents through community organizations and at parent nights at local schools and then will recruit youths once data collection for parents has been completed. The second method of recruitment for this study will be with social media sites, including Facebook, Instagram, and Twitter. Participants will be asked to click on a link in the advertisement, which will take them to the Qualtrics (Qualtrics) web page where they will fill out a form with basic demographic information (age and race) and the parents’ contact information (email and phone number). If participants meet eligibility criteria, a research staff member will contact the potential participant through email to send the information sheet for them to review and set up a time to contact them by phone to review this study and obtain verbal consent. If either the caregiver or the parent does not wish to be in this study or have their child participate in this study, no further contact will be made.

Individual interviews with parents and children will take place in 2 locations: through Zoom (Zoom Video Communications, Inc) where participants will be able to see and interact with each other, and in person with the aid of the community partners’ space. Parents and children will be interviewed separately. Members of the research team will have prolonged engagement within the community to establish trust with participants. As of December 2023, recruitment for this study began in May 2023 and is 95% completed.

#### Qualitative Interview Outcomes

Qualitative methodology allows participants to discuss their lived experiences and can account for specific details that quantitative methods may not be able to attain. The research team will conduct semistructured individual interviews. Research questions will address the factors that contribute to substance use among youths in urban cities. The interview guide will consist of questions about substance use perception and knowledge, parent-child relationships, and their perception of educational resources for Black families.

Qualitative methodology allows for participants to discuss their lived experiences and can account for specific details that quantitative methods may not be able to attain. Research questions will address the unique contextual factors that contribute to substance use in Paterson (or East Orange), challenges in discussing substance use within their families and understanding what they would like to see in a parent-child substance use prevention intervention for Black families in their community. This component of this study focuses on three specific research questions: (1) What are the social and environmental contexts of substance use initiation? (2) What are the challenges to discussing substance use within the Black family? (3) What specific components do Black families need in a substance use prevention intervention in Paterson?

In qualitative research, data collection and data analysis occur inductively through the identification of recurring themes and patterns in transcripts, field notes, and analytic memos. A thematic analysis framework will be used for this study [54]. The thematic analysis allows researchers to highlight similarities and differences across groups of participants. The research staff will work from an essentialist or realist perspective that assumes participants’ language reflects their experiences, meanings, and realities [54]. Meaningful analytical units will then be developed by using a coding scheme informed by dominant themes in the data. Topics will then be divided into several subtopics based on recurring themes within the larger topics, allowing for more in-depth analysis and complex understanding and interpretation of each theme. Each theme and subtheme will be assigned a code, and the codes will be compiled in a codebook.

A confirmability audit will be conducted where multiple coders will be used to analyze the data. Data from the interviews will first be analyzed by interviewers using open coding, whereby concepts were identified and labeled as they emerged from the data and across the interviews. Interviews will be transcribed and analyzed using NVivo (version 12; QSR International) software. The coding process will be inductive in nature and consist of categorization and grouping. Line-by-line coding will be used, and common themes will be grouped using a coding map created from NVivo to conceptualize the themes. At least a 90% interrater reliability will be achieved before codes and categories are developed. The categories that will be developed from the coding process will not be predetermined but rather formed during the coding process. After the initial coding of the data, the research team will summarize and organize the results in NVivo [55].

### Aim 2

#### Overview

We aim to adapt a family and community-based substance use prevention intervention for Black youths and families, which includes a family and community advisory board to guide the adaptation of the intervention.

#### Family and Community Advisory Board

After qualitative interviews, participants will be asked if they would like to be a member of the family-community advisory board, consisting of parents, caregivers, and leaders of family-based organizations in New Jersey. If the participant is interested, the research assistant will discuss this study with the guardian or parent and obtain informed written assent from the participants. For the focus group and individual interviews, the research assistant will discuss this study with the guardian or parent and obtain verbal assent from the participants. In describing this study to the participants, the purpose of this study will be to provide a strengths-based approach to build on the strengths of African American families and support parents and youths during the transition from early adolescence to the teen years with a specific emphasis on helping young people avoid dangerous behaviors (eg, substance use). If a participant or their guardian or parent indicates that they do not wish to participate, there will be no further involvement in this study. We will also obtain agreement from the appropriate administrator at the participating program. Findings from the qualitative study and approval from the board will aid in the adaptation of the intervention. Based on the current literature on Black families and available interventions, our study team decided to adapt an intervention that has been delivered to Black families: SAAF.

#### Intervention to Review and Adapt: SAAF Program

The SAAF program is an innovative preventative intervention program for African American youths and their families. This intervention translated research that identified racial discrimination on Black youths frequently developed into poor mental health, depression, early sexual activity, alcohol use, drug misuse, and behavioral issues [27]. Black youths who did not experience these negative health outcomes had supportive family relationships including emotional support, communicative parents, and high levels of potential control, which was identified as a protective factor [27]. While the initial SAAF program was designed for rural African American youths, it has since been successfully adapted across rural and urban settings. The intervention consists of 7 weekly gatherings lasting 2.5 hours in which caregivers and adolescents engage in discussions and activities led by community members. Caregivers focus on involved caregiving practices and providing consistent support to their adolescents. Adolescent topics include goal setting for the future, understanding who they are, dealing with early sexual desire, values, strategies for resisting peer pressure, and making good friends and choices. Caregiver topics include supporting adolescents, strict parental control, daily parenting, helping children achieve academically, encouraging racial pride, protection against negative behaviors, and maintaining adolescent-caregiver connection [28].

### Aim 3

To assess the feasibility of the intervention through theater testing, involving the family and community advisory board, we will enter step 3 of the ADAPT-ITT implementation framework.

In this aim, we will use theater testing, an innovative methodology to pretest our EBI, with the feedback of stakeholders, including this study’s team and advisory board, for intervention adaptation. Theater testing is commonly used for product testing in the areas of public service announcements; and television, video, and print advertisements [39]. ADAPT-ITT recommends no more than 5-20 participants to be involved in theater or pilot testing to receive adequate feedback and make substantive change in the intervention [52]. Out of 40 participants (20 parents and 20 children), we will invite 10-20 parent-child participants for theater testing. Participants will receive a demonstration of the product, in this case, the adapted intervention. Participants will then receive a questionnaire to provide feedback on their experience of the intervention. Documentation of participants’ interaction and reactions to information and visual materials in a constrained time frame is a significant strength of theater testing. As the theater testing population is similar to the target population, this assessment will provide accurate information about the product (intervention).

## Results

This study is part of a 2-year research pilot study award that received funding from the National Institute on Drug Abuse. Data collection for this study began in May 2023. The remaining year will focus on completing data collection, analysis, forming the family and community advisory board, and dissemination of results, and developing the intervention components. Moreover, data collection for aim 1 is 95% complete. We expect to complete all data collection for aim 1 on or before December 30, 2023. We will begin analyzing the data and consulting with our family and community advisory board beginning in February 2024. This study is funded by a research education grant from the Family Involvement in Recovery Support and Treatment Research Network, which is cofunded by the National Institute on Drug Abuse and National Institute of Neurological Disorders and Stroke (PI: AH: R24DA051946). The protocol outlines a pilot study funded through the research education grant (PI: IO). IRB approval was given in July 2022.

## Discussion

### Principal Findings

The goal of this study is to adapt a race-specific parent-child substance use education intervention for Black families living in urban cities in New Jersey which can promote family bonding, communication, and supervision, as well as acknowledge racial and ethnic-specific norms, values, and pride. The adaptation of this intervention will be co-designed based on the input of Black parents and their children and stakeholders. Through this approach, researchers will have an in-depth understanding to adapt evidence-based substance abuse prevention interventions for Black families’ parenting practices and environmental context. Other studies have demonstrated that youths report reduced use of drugs when they have positive relationships with their parents and communicate with their parents about sex and drugs [16-18]. While limited research has specifically focused on family processes involved in drug use prevention among Black families solely, we anticipate reduced rates of substance use with positive parent-child communication drugs. We anticipate that study findings will provide more context on specific barriers that Black families face in their community around preventing drug use among their children. In addition, we anticipate that study findings will enhance this study team’s knowledge of potential facilitators of substance use discussions that can be used in prevention interventions.

Further implications include an integrated community and health care provider approach to incorporate nurses within the school-based health setting and pediatric primary care settings. As nurses serve as members of one of the most trusted professions, nurses are positioned to positively influence outcomes with young Black youths as they can provide health care–related education, health promotion, prevention, and support through necessary medical treatment. By including medical and nursing professionals in this work, this project plans to take a more holistic approach to understanding the foundations of substance use and prevention in Black youths while building a community with parents and families. Nurse roles will help to expand the creation of a plan that maintains consistent outreach and sustainability by providing students with multiple opportunities to receive education and resources to integrate into the school culture.

### Study Limitations

This is a formative study using qualitative individual interviews as a methodology. A limitation of this study is that data will be collected via self-report and subject to response bias.

### Conclusions

This study aims to provide environmental level and culturally specific implications in implementation science regarding the use of adapted substance use prevention interventions guided by Black families, for Black families living in urban neighborhoods. The evidence from this study will be used for the preparation of a feasibility trial and a more robust and larger parent-children drug use prevention clinical trial specifically for Black families.

## Appendix

Multimedia Appendix 1 Reviewers summary statement.

## Abbreviations

ADAPT-ITT: Assessment, Decision, Adaptation, Production, Topical Experts, Integration, Training, and Testing
EBI: evidence-based intervention
PI: principal investigator
SAAF: Strong African American Families

## Data Availability

The data sets generated and analyzed during this study are not publicly available due to confidential reasons but are available from the corresponding author upon reasonable request.
