# Supplementary material for: Brief Parent-Child Substance Use Education Intervention for Black Families in Urban Cities in New Jersey: Protocol for a Formative Study Design
Source: JMIR Res Protoc. 2024 May 9;13:e55470. doi: 10.2196/55470 (PMC11117129; doi:10.2196/55470)
Supplement: Multimedia Appendix 1 [file resprot_v13i1e55470_app1.pdf]

Full Proposal Summary Statement: Opara
12.8.21

Significance and Innovation

Strengths

- Understanding existing gaps in knowledge about family-based preventive interventions for youth substance use and related problems among traditionally underserved and systemically oppressed Black communities is a national health priority.
- Developing racially focused substance use prevention models to improve and ultimately optimize prevention effects among Black communities is a highly significant behavioral health aim.
- Ecodevelopment theory is a rich theoretical background from which a multitude of evidence-based interventions for substance use has emerged.
- The applicant is using the Strong African American Families Program and the Family Check Up as foundations for intervention development. Both are well-researched interventions, and the foundation for adaptation is solid.
- Embedded community approaches such as that proposed here are highly promising strategies for improving community-targeted prevention programs.

Weaknesses

- The application does not always draw clean distinctions between correlational versus causal (i.e., intervention) effects when describing various aspect of substance use risk and protection. For example, the following sentence could easily be interpreted as presenting a causal relation: "…we found that parent-child communication about substance use and the parental modeling and communication of drug use within a neighborhood context, were significant factors in reducing drug use and promoting abstinence among Black girls". Yet, if in fact the data in question are correlational in nature, a more accurate description might be: "…we found that parent-child communication about substance use and the parental modeling and communication of drug use within a neighborhood context, were significant predictors of less drug use and greater abstinence among Black girls". These often-subtle distinctions assume great import in the context of compiling justification for designing a prevention model that would be tested experimentally to evaluate its causal impacts.
- The application argues that SAAF is too intensive to be implemented effectively in a large portion of families living in urban Black communities, thus requiring some degree of adaptation via ADAPT-ITT. But SAAF has the requisite evidence base and the essential focus on communication skills—so, is the deficit simply a matter of 7 sessions being too many?
- The application also asserts that briefer interventions such as the FCU have proven effective. Has the FCU produced strong outcomes in Black communities? If it has not yet been adequately tested in this context, why not simply undertake a credible test of FCU in Paterson? If there are a priori reasons for knowing or suspecting that FCU is not sufficiently tailored to be effective in supporting urban Black families, these could be elucidated. Also, the FCU is substantially different in format and content from SAAF, which may pose difficult challenges in the Decision phase.
- Although the theory is appropriate, the application does not strongly tie aims/procedures to the theoretical framework. That said, the applicant has practical experience with adapting interventions, so this is not be considered a major weakness.

Approach

Strengths

- CBPR methods are an excellent fit for conducting the proposed work.
- The PI already has a strong research-community partnership and a history of successful collaboration in the targeted community. This creates confidence that participant recruitment projections and other study goals will be ably met.
- ADAPT-ITT offers an excellent conceptual framework for guiding the intervention tailoring process in concert with CBPR methods.
- Two strong Letters of Support attest to the feasibility of achieving study aims.

Weaknesses

- The target age range for potential participants is not fully clear. SAAF focuses on youth ages 10-14, but this proposal might include 12-18? Consider selecting a narrower age range, wherein parent-youth communication is most age-salient.
- The intervention focuses on communication challenges exclusively. It might be beneficial to also gather local information about facilitators to parent-adolescent communication in this context.
- The anatomy of the interview process it not well described. From where will the PI draw the qualitative interview questions? Key details about the various aspects of data collection are scarce. For example, will dyads be interviewed separately, conjointly, or both? How long with interviews last?
- Relatedly, it is not clear what specific steps will be involved in the “theater testing” phase. It appears this step involves implementation of the entire intervention (from Latham et al, 2010), meaning delivery of SAAF and/or FCU in full. If this is correct, then it raises additional questions, including who on the team is qualified to deliver the intervention.
- The description of how the proposed analyses would contribute to a larger longitudinal ADAP-ITT process is underdeveloped. It is important, for example, to delineate how specific proposed methods would fit within each of the first 5 stages, and moreover, what the specific benchmarks for completion/success would be for each stage.
- Is the ADAPT-ITT process expected to proceed without participants being exposed directly to the content of interest? That is, there are no details about whether and how content from SAAF and FCU will be collaboratively reviewed with study participants—or if this will not occur, how the PI will draw reliable implications about intervention tailoring from the interview data.
- One issue is whether the PI can include in her motivational interviewing what families see as the goal for SU prevention in their children; measurable outcomes are often tricky to specify, and they will be needed for subsequent steps when an intervention is tested.
- The Timeline specifies Pilot Intervention and Intervention Evaluation activities, yet these appear to be ADAPT-ITT Stage 6-8 activities and thus beyond the proposed scope of work.

Investigator, Mentoring, and Productivity Plan

- Dr. Opara has an exemplary training and research background and an excellent current professional context for achieving proposed aims.
- The PI is quickly establishing herself as a valued researcher in an understudied priority area of research for the NIH. Her work is recognized by an NIH Early Independence Award in which the proposed research would be enveloped. The candidate is perfectly placed for a FIRST Research Scholar Award.
- Beyond the mentoring she will receive as a FIRST Scholar, for the next year she will participate in a R25 project designed to provide “hands on” research training to researchers of African descent.

- There is some uncertainty about the nature of original-source (i.e., model-developer) support available to the PI for implementing the ADAPT-ITT model and for adapting the SAAF and FCU models.
- Dr. Becker is ideally suited to mentor the PI in conducting the proposed work and developing a highly productive early career research trajectory. She is primed to participate in the mentoring arrangement described in the proposal, an arrangement that excellently conforms to the stipulations of the FIRST Scholars program.
- Specific end products of proposed research and mentoring activities, including an R34 application, are described and appear realistic given the scope of the proposed work.
- If study aims are achieved, study results will contribute to a strong trajectory of professional and scientific achievements within a coherent program of federally-funded prevention research.

Other

- There are several editing and consistency errors in both the PI's biosketch and main proposal. For example, in one place SAAF is listed at 9 sessions, in another at 7.
- At times the important distinction between preventive versus treatment intervention is neglected or blurred, including in the selection of references.
- There seems to be a gap from Jan '23-Sept '23, is this a planned gap in the work?
- The Budget supports a 50% RA for 12 months of work. The training, supervision, and specific activities of this person over the course of a full year are not explicated.
